# Risk Factors Associated with the Development of Immune-Checkpoint Inhibitor Diabetes Mellitus: An Integrative Review

**DOI:** 10.3390/life15071063

**Published:** 2025-07-03

**Authors:** Vivian Crowder, Veronica Brady

**Affiliations:** Health Science Center, Cizik School of Nursing, University of Texas, Houston, TX 77030, USA; veronica.j.brady@uth.tmc.edu

**Keywords:** immune mediated-checkpoint inhibitor diabetes, diabetic ketoacidosis, checkpoint inhibitors, hyperosmolar hyperglycemia syndrome, pancreatic autoantibodies, human leukocyte antigen haplotype

## Abstract

Immune checkpoint inhibitor diabetes mellitus (ICI-DM) is an emerging phenomenon in the adult oncology population, with an increased incidence reflecting the increased use of immunotherapy; however, risk factors for ICI-DM have not been fully identified. The aim of this integrated literature review was to synthesize the published literature on ICI-DM and the factors associated with an increased risk for its development. The review was guided by Sanieszko’s Epidemiology Triad theoretical framework. We conducted a literature search using the Cumulative Index to Nursing and Allied Health Literature, Web of Science, and PubMed databases. The analysis included 2030 studies that met the search criteria, 23 of which were peer-reviewed articles that met the inclusion criteria. The results demonstrated a positive relationship between older age, medical history of diabetes, the presence of susceptible alleles, and exposure to immunotherapy, with an increased risk for ICI-DM. Future studies should include larger samples, more diverse populations, and a broad range of institutions to confirm the risk factors associated with ICI-DM.

## 1. Introduction

Currently, a staggering 9 million people worldwide are living with type 1 diabetes mellitus (T1DM) [1]. The slow destruction of pancreatic cells and biological and environmental factors characterize this condition. However, symptoms do not reveal themselves until 80–90% of the beta cells of the pancreas have been destroyed [2]. Nearly 500,000 individuals are diagnosed with T1DM annually, yet definitive underlying risk factors and prevention strategies remain elusive [3]. Cancer care therapies introduced in 2011 have now led to the development of a subtype of T1DM, coined ICI-DM.

ICI-DM is an emerging phenomenon in the adult oncology population. Its classification and presentation often differ because it has an abrupt onset, and this population does not uniformly have pancreatic autoantibodies. Although this phenomenon has been described in the literature since the dual use of immunotherapy in the oncology population began in 2011, definitive risk factors associated with the development of ICI-DM require further study [4]. This review aimed to synthesize the published literature on ICI-DM to clarify what is known about the risk factors associated with the development of ICI-DM among adult oncology patients and to identify areas needing further investigation. Synthesis of current literature can lead to a better understanding of the factors associated with the development of ICI-DM and thus increase the measures to proactively identify those at risk.

## 2. Background and Significance

ICI-DM is characterized by abrupt destruction of the beta cells of the pancreas, leading to the development of insulin-dependent diabetes [5]. This condition results from the use of immunotherapy for various malignancies and was first reported among patients with malignant melanoma [6]. Immunotherapy is categorized into various classifications; the classifications associated with the development of ICI-DM include programmed cell death protein-1 (PD-1) and programmed death ligand (PD-L1) [7].

Immunotherapy aids in the destruction of cancer cells by upregulating the immune system to attack cancer cells [8]. For some, this enhanced immune response has adverse effects on non-targeted organs, resulting in immune checkpoint-related complications, such as ICI-DM [4]. The development of ICI-DM requires providers to act promptly to identify this life-threatening condition, which requires lifelong treatment with insulin. Because current evidence demonstrates a weak association with risk factors for ICI-DM, no preventive strategy is currently in place to protect against the development of this condition.

The incidence of ICI-DM has steadily risen. Earlier estimates suggested that approximately 1% of the oncology population developed this life-threatening condition [4]; however, recent studies report that the incidence has increased to 3.5% with expanded use of immunotherapy [9]. The development of ICI-DM and concurrent cancer can lead to a decline in the overall quality of life in this population, resulting in higher rates of psychological and emotional distress and a lack of confidence [10]. Furthermore, the cost associated with a diagnosis of T1DM is staggering. Persons with newly diagnosed diabetes account for one of every four healthcare dollars and a total estimated financial cost of USD 412.9 billion annually, of which USD 106.3 billion is associated with lost productivity from diabetes-related complications resulting in unemployment in the adult population [11].

Some risk factors that have been associated with ICI-DM include exposure to immunotherapy, solid tumor diagnosis, T-cell expression in the pancreas, and human leukocyte antigen (HLA) genotyping, which is measured by evaluating the DR3, DR4, and DR9 haplotypes, with A2 being the dominant HLA serotype [5,8,12,13]. However, these findings are not conclusive, and there is a lack of consensus regarding the demographic, biological, genetic, and environmental factors associated with ICI-DM development [12]. Identifying these factors can help preemptively recognize at-risk individuals and streamline active surveillance strategies for this population, thereby mitigating the adverse events associated with ICI-DM development.

### Theoretical Framework

**Figure 1:**
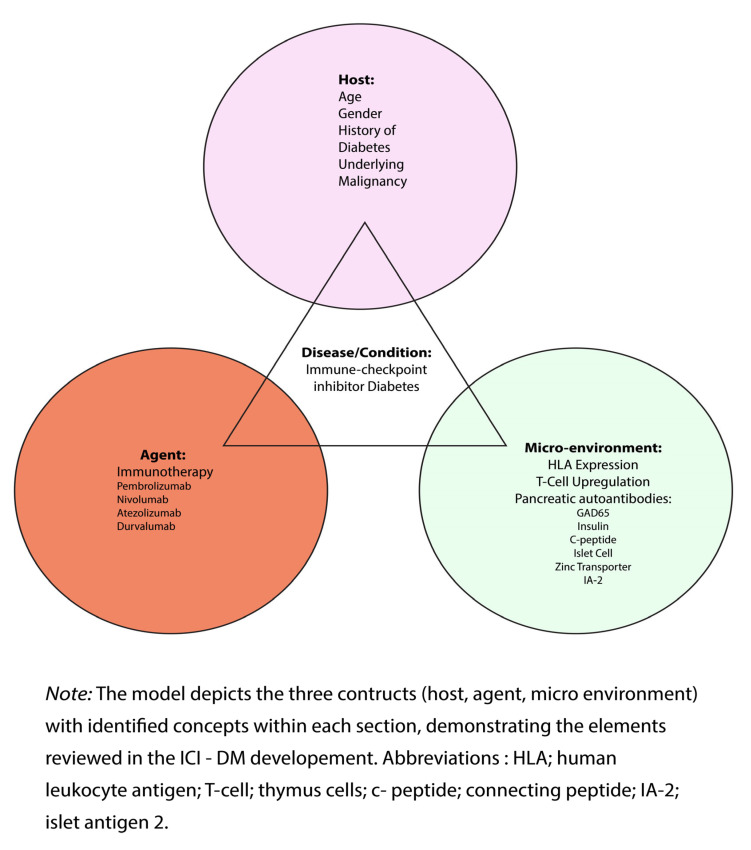
**Modified Epidemiology Triad**.

This review was guided by Snieszko’s Epidemiology Triad [14], which was described by Van Seventer and Hochberg [15]. This classic theoretical framework describes infectious disease causation by displaying the relationship between the agent (pathogen), host, and environmental factors. Although originally developed for infectious diseases, this framework was modified to illustrate the potential factors associated with ICI-DM and was used to guide the data extraction for this review (Figure 1).

According to this framework, the risk of developing ICI-DM depends, in part, on the characteristics of the *host*, making them more susceptible to ICI-DM. Existing literature suggests that the susceptible host is female with a history of melanoma, non-small cell lung cancer (NSCLC), or renal cell carcinoma (RCC) [7,12]. Additional host factors include younger age, a median age of 66 years +/− 5 years, and pre-existing diabetes [6].

*Agent factors:* The original framework describes exposure to an agent that leads to the development of a specified condition. For this review, the agent is immunotherapy, which leads to the development of ICI-DM. The development of ICI-DM is associated with the introduction of immunotherapy, most notably PD-1 and PD-L1 agents, and is seen after the first three cycles of treatment or within 9 weeks of immunotherapy exposure [4,5,6,7,12]. Once a susceptible host is exposed to one of these agents, the risk of developing ICI-DM increases by up to 1%.

*Micro-Environment:* In the original framework, environmental factors were outlined to depict the relationship between constructs and disease development. The micro-environment was described as external environments, such as social, behavioral, and economic factors [15]. For this review, the micro-environment was adapted to reflect the internal factors associated with ICI-DM development. When a susceptible person is exposed to certain agents, small changes in their body’s environment can trigger the immune system to attack the pancreas. This includes the appearance of autoantibodies like glutamic acid decarboxylase 65 (GAD65), destruction of islet cells, and the loss of insulin antibodies, C-peptide, zinc transporters, and insulin-associated protein [3,4,5,13,16]. Additional micro-environmental elements were identified and thought to play a role in developing this condition, including HLA expression and T-cell upregulation, rendering the pancreas more susceptible to destruction from the immunotherapy agent [16]. The concepts associated with the micro-environment describe the impact of immunotherapy on the host’s internal immune regulatory system, rendering the host more vulnerable to ICI-DM.

*Disease:* ICI-DM is at the center of the framework and is the outcome of interest. The triangle represents the relational constructs of host, agent, and micro-environmental factors identified as potential risk factors for ICI-DM (Figure 1).

## 3. Methods

### 3.1. Data Sources and Searches

With the assistance of a medical librarian, we conducted a search of the literature published from 2014 to October 2024, reflecting the timing of immunotherapy toxicity publications. To ensure that a full scope of literature related to ICI-DM risk factors was captured, we searched the Cumulative Index to Nursing and Allied Health Literature (CINAHL), Web of Science, and PubMed databases. The initial literature search was limited to full-text articles available in English. Given that published manuscripts are not uniform in the identification of ICI-DM, a combination of Medical Subject Headings (MeSH) and Boolean operators were employed to capture this phenomenon in CINAHL and PubMed using the following search terms: “immunotherapy induced diabetes AND risk factors,” “immune checkpoint inhibitors AND diabetes,” (TI (diabetes OR diabetic)) AND (TI (immunotherap* OR checkpoint inhibitor*)), “diabetes mellitus type 1,” “diabetic ketoacidosis,” “immunotherapy,” “checkpoint inhibitor,” “ICI” “nivolumab,” “pembrolizumab,” “PD-1,” “PD-L1,” and “risk factors”.

### 3.2. Inclusion and Exclusion Criteria

Articles were included in the review if they (a) included adults ≥ 18 years old with a primary aim of oncology population receiving immunotherapy, (b) were published in English, (c) reported an intervention that included checkpoint inhibitors, (d) measured ICI-DM as an outcome, and (e) were published within the past 10 years. Reports were excluded if they (a) included a pediatric population, (b) reported an intervention administering CAR-T cells, T-cell infusions, or any vaccine, (c) measured outcomes, including type 2 diabetes mellitus (T2DM), juvenile diabetes mellitus, COVID-19, and HIV, or (d) were a systematic or narrative review or case report. To evaluate the quality of the studies included, the Critical Appraisal Skills Programme for case-control, cohort, and observational studies was used [17,18,19,20,21]. Because this review aimed to understand the current risk factors associated with ICI-DM, no studies were excluded because of a lack of rigor. A two-person rater agreement was performed by V.C. and V.B. The variables extracted for this review included an analysis of the study design, population characteristics, and an assessment of outcome criteria. To minimize selection bias, each reviewer performed an independent evaluation of the identified studies. Any discrepancies were resolved by the primary author. An online tool, Covidence, which is a web-based collaboration software platform that streamlines the production of systematic and other literature reviews, was used to screen the titles/abstracts and full-text articles (2023).

## 4. Results

**Figure 2:**
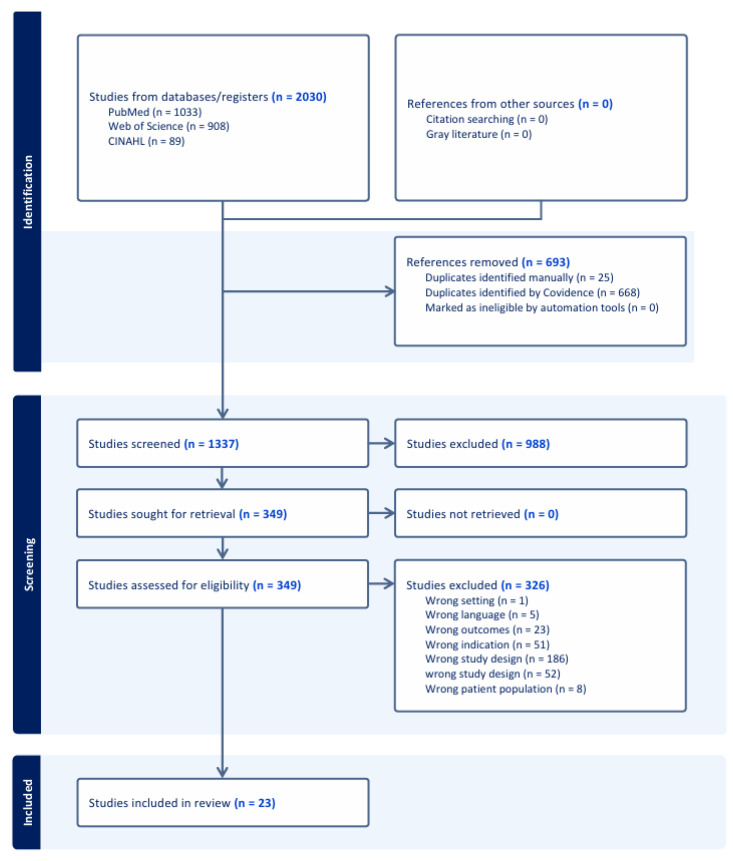
Prisma diagram.

A total of 2030 studies met the search criteria. After removing 693 duplicates, we screened 1337 studies by their titles and abstracts. Thereafter, 988 studies were deemed irrelevant, leaving 349 studies for full-text review. Ultimately, 23 articles met the inclusion criteria and were included in our review (Figure 2). These studies were imported in Covidence.

### 4.1. Study Characteristics

A total of 23 articles were included in the review (Table 1). The studies were an accumulation of case-control series [22,23,24,25] and prospective analyses [26,27,28,29,30,31], with the majority being retrospective analyses [32,33,34,35,36,37,38,39,40,41]. The studies were conducted in diverse countries, including China, Japan, Hong Kong, Australia, Korea, the United Kingdom, and the United States. Each study addressed at least one aspect of the conceptual framework (Table 2); however, seven studies addressed all constructs of the framework [25,28,30,33,34,35,39,42], providing a complete analysis of the factors influencing the risk of ICI-DM. Of the studies reporting ICI-DM incidence, 11 reported less than 1% in their cohort [6,13,22,23,26,28,29,34,35,36,37,40]. However, 4 studies reported an incidence higher, averaging 1.2% [33,39,41,42]. Most studies reported ICI-DM presentation 3–4 months after onset. However, some studies reported a more delayed onset of up to 2 years [22,23,25,34].

#### 4.1.1. Host Characteristics

The mean age at the onset of ICI-DM was 63 years [22,23,24,27,28,30,31,32,33,35,36,37,38,39,42]. However, a few studies reported the average age to be nearer in the early ’70s [6,25,26,29]. Gender analysis revealed that males had a higher risk of ICI-DM, whereas other studies did not report this association [23,27]. A history of diabetes was reported in 11 studies [22,26,28,32,33,36,37,38,39,41,42], of which three identified pre-existing T1DM or T2DM as positively correlated with the development of ICI-DM [6,13,33,34]. The solid tumor diagnoses in the study were diverse and included metastatic melanoma, NSCLC, RCC, urethral carcinoma, and cancers of the genitourinary system, breast, gastrointestinal tract, and head and neck [40].

#### 4.1.2. Agent Characteristics

The results regarding which agents were positively correlated with ICI-DM development were conflicting. Three studies reported that the combination of CTLA-4 and PD-1/PD-L1 was positively correlated with the development of ICI-DM [6,13,36]. However, three other studies found that PD-1/PD-L1 agents were strongly correlated with ICI-DM development [27,39,41]. Two other studies did not report a significant difference between the immunotherapy agents and the risk for the development of ICI-DM, but did mention that the onset of ICI-DM was earlier in those receiving combination treatment than in those receiving single-agent PD-1/PD-L1 [32,35].

#### 4.1.3. Micro-Environment Characteristics

##### HLA-Expression and T-Cell Upregulation

HLA haplotypes are typically seen in the development of T1DM. In the studies in which HLA protective and susceptible alleles were assessed, class II HLA-DRB1 expression was associated with an increased risk of total insulin loss (T1DM) (20–70%) [24,28,30,33,34,39,42]. One study found that class I HLA alleles were also associated with an increased risk of total insulin loss (T1DM) [35]. Other studies found that HLA-DRB1 haplotypes were protective against insulin deficiency, but at a much lower frequency than the susceptible allele [30,39,42]. Another study reported the detection of an allele that protected against the development of concurrent ICI-DM and immune checkpoint inhibitor-isolated adrenocorticotropic hormone deficiency [31]. No studies have analyzed the relationship between T-cell lymphocytes and the development of ICI-DM.

##### Pancreatic Autoantibodies

The total number of patients across all studies who developed ICI-DM or unexplained new-onset diabetes was *n* = 1185 out of a sample population of N = 61,003. Of this sample population, pancreatic autoantibodies were detected in 0.07% of cases, 90 of which were GAD65 [24,28,31,32,33,34,35,36,37,38,39,40,41]. Islet antigen antibodies were detected in 25 individuals [22,24,28,30,31,36,38,41]. In one study, insulin antibodies were detected in 11 individuals, and zinc transporters were detected in 2 individuals [36]. None of the other studies detected zinc transporters within their populations. Of the studies evaluating C-peptide production after new onset ICI-DM, all reported individuals had either an inappropriately low C-peptide level in the setting of hyperglycemia or undetectable levels [13,22,24,25,28,30,32,33,34,35,36,37,38,39,40,42].

## 5. Discussion

The included studies elucidated the various risk factors associated with ICI-DM development. Studies that included people with a medical history of diabetes noted that this condition was positively correlated with the development of ICI-DM [13,28,33,40]. However, this correlation was not established in all studies, as many excluded people with pre-existing T2DM and T1DM. Hence, future studies should focus on this association to determine whether pre-existing diabetes is a risk factor for ICI-DM development or whether those with pre-existing diabetes progress to ICI-DM faster. Two studies identified male gender as a factor; however, this finding could reflect selection basis, given that one study included only males [23], and in another study, over 65% of the sample were males [27]. The underlying malignancies were widely representative of many solid tumor diagnoses, reflecting the type of malignancies for which immunotherapy was first approved; therefore, higher rates of ICI-DM would be expected in this population, particularly those with melanoma, RCC, and NSCLC. Some studies included liquid tumor diagnoses such as lymphoma [24,30,41], but the small sample sizes made drawing conclusions difficult.

The relationship between the development of ICI-DM and immunotherapy agents was clear: most studies reported the frequent association of PD-1 and PD-L1 agents with the development of ICI-DM, and combination treatment was associated with higher risk. Therefore, given the known risk associated with PD-1 and PD-L1 agents, oncology providers should be sure to educate recipients of this treatment and its potential risks.

Micro-environment characteristics are an emerging area that could assist in the proactive identification of individuals with traits associated with an increased risk for ICI-DM. Recent studies have identified HLA haplotypes for susceptible and protective alleles akin to T1DM, identified in persons who develop ICI-DM [24,28,30,33,34,39,42]. Additionally, micro-environment characteristics, such as GAD65, insulin antibodies, zinc transporters, and islet cell antibodies, are infrequently detected in persons with ICI-DM and, therefore, did not appear useful in proactively identifying persons at high risk. C-peptide is consistently low or undetectable in those with ICI-DM; therefore, exploring timed testing to assess the rapidity of decline could be beneficial for detecting early ICI-DM, but it is not a marker that can be used to identify high-risk individuals.

### Strengths and Weakness

This review highlights future directions for the assessment of patients receiving immunotherapy to proactively identify those at increased risk for ICI-DM. Although not consistently included in each study, factors associated with an increased risk of ICI-DM include a medical history of diabetes and the presence of HLA haplotype alleles susceptible to T1DM. The identification of these potential risk factors is paramount for this population and provides guidance for the development of screening algorithms for individuals identified as being at risk.

Twelve of the studies were retrospective analyses, thus limiting the researchers’ ability to control for confounding factors such as steroid administration and the ability to proactively assess for micro-environment characteristics used to identify risk factors for ICI-DM. Furthermore, 10 studies evaluated HLA characteristics, which have shown promise in identifying high-risk individuals, but additional studies are needed to confirm this relationship. Much of the cohort was unequally representative of the general population, and many of the studies were performed at single institutions. This review was limited to studies in English, which may have introduced language bias and affected our findings. This decision was based on the feasibility constraints of the authors. This limitation should be considered when interpreting the generalizability of our results. Several studies based in Asia comprised only various Asian demographics, and the U.S. studies had larger cohorts of White individuals. Also, inadequate sample size was a limitation in many of the studies that assessed only persons who developed ICI-DM rather than analyzing an entire cohort receiving immunotherapy. Potential confounders were not consistently addressed; for example, glucocorticoids were not consistently addressed in the included studies. We did not conduct a formal sensitivity analysis based on study quality. However, we acknowledge that variability in methodological rigor may have impacted the overall conclusions. We also recognize the potential for publication bias, as ICI-DM may be underreported, skewing the actual incidence risk patterns associated with immune checkpoint inhibitors.

## 6. Conclusions

Immunotherapy has changed the prognosis for previously terminal malignancies by improving overall life expectancy, but it is not without risk. Immunotherapy has been associated with developing ICI-DM, a subtype of T1DM, given its hallmark of absolute insulin deficiency. To date, no identifiable factors have helped identify high-risk individuals and prevent this life-altering condition. This review aimed to elucidate what is currently known about people who develop this condition and summarize potential factors associated with high-risk individuals. The findings of this review suggest that HLA haplotypes for susceptible traits, medical history of diabetes, and exposure to PD-1 or PD-L1 place individuals at higher risk. Future studies should explore how data mining, machine learning, and artificial intelligence-based predictive models could enhance the identification and risk stratification of ICI-DM.

## Figures and Tables

**Table 1:** Study characteristics.

Author/Country	Aim/Design	Sample Size/Setting	Incidence/Onset	Findings	Strengths	Limitations	Study Quality Assessment	**Corresponding Reference**
Stamatouli et al., 2018 United States	(PA) Identify attributes that might lead to clinical insights into ICI-DM.	N = 2960 (27 with ICI-DM) Endocrinology at Yale New Haven Hospital and University of California	(I) 0.90% (O) 20 (W)	Time of DM onset can be long after the initial CPI treatment. The presence of preexisting T2DM does not preclude the development of ICI-DM. There are clinical and laboratory features of this form of DM that are like but also clearly different from spontaneous T1DM.	Compared non-ICI-DM population with exposure group. HLA typing reveals striking information that will inform future studies.	Homogeneous population making it not very generalizable.	91% CASP Cohort Study	[28]
Kotwal et al., 2019 United States	(RA) Characterize potential predictors of ICI-DM.	N = 1444 (21 with ICI-DM) Mayo Clinic, Single Institution	(I) 1.40% (O) Median 4–5 months	ICI-DM occurred most frequently with pembrolizumab (2.2%) compared with nivolumab (1%) and ipilimumab (0%). The median age was 61 years, and body mass index was 31 kg/m^2^, which are both higher than expected for spontaneous T1DM. The most immune related event was thyroid disease. New-onset ICI-DM developed after a median of four cycles or 5 months; 67% presented with diabetic ketoacidosis and 83% with low or undetectable C-peptide. Autoantibodies were elevated in 5/7 (71%) pts at the time of new-onset diabetes. DM did not resolve during a median follow-up of 1 year.	Large cohort sample. Generalizable to other populations. Excluded persons with preexisting T1DM and persons exposed to glucocorticoids.	Retrospective. Single institution. No pretest of the biomarkers for antibodies prior to the initiation of ICI.	100 % CASP Cohort Study	[41]
Marchand et al., 2019 Europe	(PA) Describe both pancreatic functions, immunological features and change in pancreas volume in subjects ICI-DM.	N = 6 pts with ICI-DM Single institution	(I) -- (O) 4 (M)	ICI-DM was not associated with T1DM-related autoantibodies.	Immunological analysis of persons with ICI-DM along with pancreatic volume analysis.	Small sample size. Homogeneous sample. Selection bias.	83% CASP Cohort Study	[30]
TsangVHM et al., 2019 Australia	(RA) Describe the nature of ICI-DM, and potential immunological and genetic predictive factors.	N = 538 pts of which 10 developed ICI-DM Melanoma Institute Australia and the Department of Endocrinology, Royal North Shore Hospital, Sydney, Australia	(I) 1.90% (O) 25 (W)	ICI-DM occurs within 63 weeks of starting anti-PD-1 therapy with rapid decline in C-peptide concentrations consistent with sudden b-cell failure. -Most pts do not have DAAs, and several have HLA class II haplotypes that normally protect against T1DM. CIADM is distinct from latent autoimmune diabetes of adult onset, in which positivity for DAAs is associated with slow progression of beta-cell failure. The sudden loss of C-peptide and insulin in the context of immunotherapy is a marker of CIADM.	Baseline samples of BG, hemoglobin A1c, insulin, and C-peptide were measured in plasma samples before the first dose of immunotherapy and for up to 35 months after diagnosis of ICI-DM. Large sample size.	Lack of identification of race of sample group. Only evaluated the melanoma population, which can alter some results when transcribed to other malignancies.	85% CASP Cohort Study	[42]
Byun et al., 2020 United States	(RA) Characterize ICI-DM in a single-institution case series.	N = 18 pts with ICI-DM Memorial Sloan Kettering Cancer Center	(I) 0.37% (O) 3.65 (M)	Nine pts presented with DKA, and nine presented with hyperglycemia without DKA. The median initial BG was 27.92 mmol/L. There was no apparent time to IDDM differences between groups receiving combination ICI therapy versus monotherapy. Autoimmune toxicity from ICI may be associated with improved cancer-specific mortality, as an overly robust immune system could lead to increased antitumor efficacy.	Large oncology population receiving immunotherapy -Removed pt with pre-existing diabetes. Two-tailed Wilcoxon rank-sum testing compared pt characteristics. Overall survival was estimated using Kaplan-Meier methodology.	Lack sufficient power to detect significant subgroup differences. Small sample size.	83% CASP Cohort Study	[35]
Knight et al., 2021 UK	(PA) Determine the frequency and clinical presentations of immune-mediated endocrinopathies in pts presenting as emergencies.	N = 648 pts treated with ICIs (4 with ICI-DM) Oncology Emergency Center, Single Institution	(I) 0.58% (O) 8 (C)	Presentations to emergency settings with irAE. Early recognition of immune-mediated toxicities is important.	Large population sample. Included persons with and without ICI toxicity. Findings are consistent with the current literature.	Confounding factors were not addressed such as steroids or underlying conditions like diabetes. Pancreatic enzymes needed for diagnosis ICI-DM were not mentioned. Single center evaluation.	83% CASP Cohort Study	[29]
Leiter et al., 2021 United States	(RA) Characterize the prevalence and factors associated with hyperglycemia in pts treated with ICIs.	N = 385 total with 48 having new onset hyperglycemia after ICI-DM NCI-designated cancer center	(I) 0.03% (O) 9.7 (W)	New hyperglycemia in pts receiving ICIs was mostly related to glucocorticoids. A small pt subset had new unexplained hyperglycemia, suggesting that ICIs might have a role in promoting hyperglycemia.	Large sample size. Analyzed all pts treated with ICIs. Included a diverse pt population relative to clinical trials.	Retrospective. Limited analysis of A1C. No antibody testing uniformly collected on the cohort of pts who developed hyperglycemia that was not explained by other causes such as steroids. Lack of clear ICI-DM in this study.	50% CASP Cohort Study	[37]
Basak et al., 2022 The Netherlands	(Nested Case-Control) Investigate C-peptide levels as a potential predictor of ICI-DM and describe the presence of islet autoantibodies and course of pancreatic enzymes in pts with and without ICI-DM.	N = 1318 pts (10 pts with ICI-DM 2 pts excluded due to primary endpoint serum data not collected before developing ICI-DM.) Erasmus University Medical Center and Amphia Hospital	(I) 0.70% (O) 6.9 (M)	-No substantial difference in C-peptide levels or course during therapy. C-peptide course before the onset of ICI-DM is not a predictive biomarker for the onset of this toxicity. Routine measurements of islet autoantibodies before or during therapy might not be useful for predicting or early detection of ICI-DM.	Matched case with controls who received the same type of treatment.	This study was not powered to perform statistical analyses. Overrepresentation of PD-1 monotherapy, which could have influenced the outcomes. Confounding factors such as steroids were not considered.	72% CASP Case Control Studies	[22]
Chen et al., 2022 United States	(RA) Understand clinical risk factors for ICI-DM and its impact on survival in pts.	N = 30,337, of which 261 developed ICI-DM De-identified cohort of Optumae Clinformatics Data Mart, which captures a privately insured population from a diverse group of health plans in the U.S.	(I) 0.86% (O) 10 (W)	Dual use of immunotherapy (CTLA-4) and (PD-1) or (PD-L1) was associated with increasing risk of ICI-DM. Younger age and pre-existing non-T1DM are also associated with a higher risk of ICI-DM. Prior use of immunosuppressive medications was associated with a lower incidence of ICI-DM. The development of ICI-DM does not seem to impact pt survival significantly.	Large sample. Generalizable to the population given that they evaluated based on U.S.-wide insurance ICD codes.	Dependence on ICD-10 codes. Lack of information on severity of adverse events and tumor stage. Unable to study the effect of ICI-DM on patient survival. Unable to identify if the significance association between prior use of immunosuppressants and poor survival is because of medication or due to the underlying autoimmune disease.	100% CASP Cohort Study	[6]
Inaba et al., 2022 Japan	(PA) Explain risk factors for ICI-DM.	N = 871 patients (7 patients total developed ICI-DM) Japanese Red Cross Society Wakayama Medical Center, Wakayama Medical University Hospital, and Nagoya University	(I) 0.80% (O) 2–21 (C)	HLA-DPA1*02:02 and DPB1*05:01 alleles were observed in most of the pts. Allele frequencies were significantly higher than those in ICI controls and controls of the Japanese general population. HLA-DRB1*04:05 allele frequencies were significantly higher than those in the general population.	Ability to find a linkage between HLA and the development of ICI-DM. Power analysis calculation. Able to analyze the population who did and did not develop ICI-DM.	Statistical techniques such as multiple regression analysis to identify the association of the HLA risk alleles and haplotypes with blood biomarkers could not be conducted because of the insufficient number of pts who developed ICI-DM.	100% CASP Cohort Study	[25]
Muniz et al., 2022 Canada	(RA) Describe the characteristics of ICI-induced IDDM to understand their tumor response rates and survival.	N = 34 Five academic Canadian cancer centers	(I) -- (O) 2.4 (M)	In pts treated with PD-1/PD-L1, onset of ICI-DM was ~3 months. In pts treated with PD + CTLA-4, onset of ICI-DM was ~1.4 months.	Multicenter approach. ICI-induced IDDM occurs acutely and may be potentially fatal. -ICI-induced IDDM is triggered by a blockade of the PD1/PD-L1 axis.	Retrospective design. Missing demographic data (race). Lack of HLA genotyping. - Only analyzed persons with ICI-DM	83% CASP Cohort Study	[32]
Chan et al., 2023 Hong Kong	(PA) Analyze risks of pts receiving PD-1 and PD-L1 agents.	N = 3375 pts were analyzed (new-onset DM occurred in 457 pts) 13.5% Data were extracted from the Clinical Data Analysis and Reporting System, a prospective, population-based electronic medical database.	(I) 8.6 cases per 100 person-years (O) --	Users of ICI may have a substantial risk of new-onset DM. Risk for ICI-DM may be higher in males but did not differ between PD-1i and PD-L1i. DM occurred in 306 PD-1i users (12.6%) and 85 PD-L1 users. No difference in the risk of new-onset DM between PD-1i and PD-L1i.	Analyzed data from a population-based database that included all pts who have ever received any ICI.	Omitted pts receiving CTLA-4 inhibitors in the overall analysis. Did not specifically analyze ICI-DM. Due to the nature of the database used, cancer staging and histological subtypes were not available. Confounders not considered.	42% CASP Cohort Studies	[27]
Inaba et al., 2023 Japan	(Case Control) Study novel amino acid polymorphisms in HLA class II molecules in pts with ICI-DM, to help predict the development of ICI-DM	N = 47 pts (12 who developed ICI-DM) Japanese Red Cross Society Wakayama Medical Center, Wakayama Medical University Hospital, and Nagoya University	(I) -- (O) 9–121 (W)	HLA-DP5 as a predisposition molecule was established, and significant amino acid polymorphisms at HLA-class II molecules in pts with ICI-DM. Conformational changes in the peptide-binding groove of the HLA-DP molecules may influence the immunogenicity of proinsulin epitopes in ICI-DM.	Power analysis. Detailed analysis of HLA binding.	Lack of clear matching with controls. Small sample size.	72% CASP Case-Control Study	[25]
Iwamoto et al., 2023 Japan	(RA) Evaluate the incidence of endocrine-related irAEs.	N = 466 pts (5 pts with ICI-DM) Kawasaki Medical School Hospital	(I) 1.10% (O) 177.4 (D)	Endocrine-related irAEs diagnosed by blood tests were correlated with survival. Pts with a history of T2DM are more likely to develop IDDM The prevalence of anti-GAD antibodies was 51% (13), but only 1 among the 5 pts in this study.	Analysis of multiple endocrine irAE. Large sample size.	Single-center, retrospective, observational study. Homogenous population. Endocrine testing was not performed in all pts.	66% CASP cohort study	[33]
Jeun et al., 2023 United States	(RA) Characterize clinical outcomes and survival in melanoma pts with ICI-DM.	N = 76 pts with ICI-DM The University of Texas MD Anderson Cancer Center	(I) -- (O) 12.5 (W)	Pts who had ICI-DM were younger, and a higher percentage were undergoing combination therapy with anti-CTLA4 and anti-PD-1 agents than pts in the control group.	Single largest cohort of pts with ICI-DM. Provided novel information on the use of diabetes technology in this pt population and highlighted the risk of readmissions and BG variability in these pts.	Retrospective. Limited to survival comparison in the melanoma cohort due to lack of access to clinical databases for other cancer subtypes.	91% CASP Cohort Study	[36]
Kawata et al., 2023 Japan	(Case Control) Evaluate pancreatic histological findings of ICI-DM compared with those of pts who received ICI therapy but did not develop ICI-DM.	N = 11 3 ICI pts, 3 non-T1DM pts, and seven controls. Osaka University	(I) -- (O)12 (W)	Confirmed the depletion of beta cell area, the increase of alpha-cell area, and the infiltration of macrophages as well as T lymphocytes to and around the islets in the ICI-DM pts. Absence of PD-L1 expression on residual beta and alpha cells in these pts.	Power analysis. Evaluation of HLA typing in pancreatic tissue.	Small sample size. Study included surgical cases and autopsy cases. Pancreatic tissues of autopsy cases may be less stained than those of surgical cases; the results should be interpreted with caution. No analysis of monoclonality of T-lymphocytes infiltrating into the pancreas in both ICI-DM pts and non-T1DM pts.	28% CASP Case Control Study	[24]
Lee et al., 2023 Korea	(Case Control) Compare the risk of new-onset DM between pts receiving an ICI and those receiving CC.	N = 1326 total, but 221 received ICI therapy of whom 27 pts developed ICI-DM) Tertiary care hospital database at Severance Hospital	(I) 0.12% (O) 17–56 (C)	ICI therapy is associated with an increased risk of incident diabetes compared with CC. The BG levels of pts treated with an ICI, especially males and those with prominent lymphocytosis after ICI treatment, need to be monitored regularly to detect ICI-DM as early as possible.	Case control matched age, sex, and cancer type to the ICI group. All pts receiving steroids were excluded. Trajectory approach was performed with a collection of demographic and laboratory data, allowing investigation on whether the trajectory cluster with an increasing BG pattern in the ICI group had distinguishable clinical characteristics.	Retrospective analysis. Trajectory changes in insulin resistance and secretion were not investigated. No reporting of how the diagnosis of ICI-DM is concluded and confirmed.	81% CASP Case Control Study	[23]
Liu et al., 2023 China	(Cohort Study) Investigate the clinical characteristics and HLA genotypes of pts with ICI-DM	N = 74 pts with (23 having ICI-DM) Third Xiangya Hospital of Central South University	(I) -- (O) 5 (C)	ICI-DM shares similar clinical features with T1DM, such as acute onset, poor islet function, and insulin dependence. Marked differences in T1DM, the lack of islet autoantibodies. Low frequencies of HLA typing T1DM susceptibility and high frequencies of protective HLA haplotypes.	Strong comparison of T1DM with persons with ICI-DM. Clean data collection with antibodies collected on 98% of pts. Statistical and clinical significant information.	Small sample size. Single institution. Homogeneous population.	91% CASP Cohort Study	[39]
Zhang et al., 2023 United States	(RA) Report the incidence and characteristics of new onset and worsening of DM in pts treated with ICIs.	N = 2477 (25 with ICI-DM) Roswell Park Comprehensive Cancer Institution	(I) 1.01% (O) 12 (W)	The incidence of ICI-DM or worsening of DM is 1.01%. ICI-DM is usually associated with the use of PD-1 or PD-L1 checkpoint inhibitors and not anti- CTLA-4 therapy. Among the 14 pts who developed new onset ICI-DM, 93% received a PD-1 checkpoint inhibitor and 7% received a PD-L1. 3 pts received CTLA-4 as a combination therapy.	Large cohort over 10 years of data. Appropriate exclusion of pts on glucocorticoids.	Retrospective. Lack of comparison of incidence of DM in a cohort of oncological pts treated vs. not treated with ICIs. Not all pts assessed for pancreatic antibodies, which would have helped inform the current body of literature on ICI-DM.	100 % CASP Cohort Study	[38]
Akturk et al., 2024 United States	(RA) Investigate whether routine monitoring of BG can predict the onset of hyperglycemia associated with ICI-DM.	N = 89 adults (13 with ICI-DM) Single center	(I) 0.15% (O) 3 (M)	Greater than 70% of pts develop ICI-DM in the first 90 days after the first dose. Routine monitoring of BG at ICI infusion visits does not predict the rapid onset of hyperglycemia associated with ICI-DM but could be beneficial for pts at high risk, such as those having HLA-DR4.	Large longitudinal data set of BG with a comparison of two groups (ICI-DM and ICI with no T1DM). There is a need to monitor BG levels more vigilantly.	Retrospective. Variable exposure to glucocorticoids not identified.	91% CASP Cohort Study Checklist	[33]
Elshafie et al., 2024 Oman	(RA) Determine characteristics and management of ICI-related endocrinopathies.	N = 139 pts (1 pt developed ICI-DM) Sultan Qaboos Comprehensive Cancer Care and Research Centre	(I) 0.70% (O) 3 months	Pts diagnosed with genitourinary cancers had a significantly higher risk of developing endocrine irAEs. The presence of any comorbidity versus no comorbidity was a significant negative predictor of toxicity. A higher disease stage (namely, stage IV) did not predict toxicity. Prior history of treatment, including surgery, radiation, or chemotherapy, was not associated with toxicity.	Focused oncology center population. Ability to review multiple irAE related to endocrine. -Study of the entire oncology population instead of focusing only on the group that developed adverse events.	Retrospective. Absence of standardized testing protocol for assessing endocrine adverse effects. Single center. Small sample size.	87% CASP Cohort Studies	[40]
Ono et al., 2024 Japan	(PA) Analyze and compare HLA signatures associated with ICI-DM and ICI-IAD in pts with both conditions.	N = 47 (33 with ICI-DM) Single Center	(I) -- (O) 22–29 (W)	DQA1*03:02, and DRB1*13:02-DQB1*06:04 were considered susceptible HLAs DRB1*15:02-DQB1*06:01 was considered a protective HLA. In pts with ICI-IAD, DRB1*15:02-DQB1*06:01, is considered a susceptible HLA. Given that DRB1*15:02-DQB1*06:01 was not detected in pts with ICI-DM/IAD, the presence of the DRB1*15:02-DQB1*06:01 haplotype appeared to protect against the co-occurrence of T1DM in pts with ICI-IAD.	Power analysis. HLA analysis in the ICI-DM group, along with complete pancreatic analysis from the entire sample.	-Small sample size. Missing data from some HLA analysis. Homogeneous sample. Lack of comparative assessment with persons without ICI-DM who are exposed to immunotherapy.	83% CASP Cohort Study	[31]
Ruiz-Esteves et al., 2024 United States	(RA) Define incidence, risk factors, and clinical spectrum of ICI-DM.	N= 14,328 (64 with ICI-DM) Mass General Brigham	(I) 0.45% (O) NR	Preexisting T2DM and treatment with combination ICI were risk factors of ICI-DM. T1DM was associated with ICI-DM risk, demonstrating a genetic association between spontaneous autoimmunity and irAEs. Pts with ICI-DM were in 3 distinct phenotypic categories based on autoantibodies and residual pancreatic function, with varying severity of initial presentation.	Large cohort with heterogeneous population. Multiple malignancies represented. Compared persons with and without ICI-DM.	Retrospective. Not able to determine how combination chemotherapy/ICI regimens may be associated with the incidence of ICI-DM. Genetic data reflect pts who were included in the PROFILE study and not the full cohort, posing selection bias.	91% CASP Cohort Study	[13]

Note. Abbreviations: ICI-DM—immune-checkpoint inhibitor diabetes mellitus, RA—retrospective analysis, PA—prospective analysis, W—weeks, M—months, D—days, C—cycles, T1DM—type 1 diabetes mellitus, BG—blood glucose, IDDM—insulin-dependent diabetes mellitus, NR—not reported, CC—conventional chemotherapy, pts—patients, irAE—immune-mediated adverse effects, HLA—human leukocyte antigen, ICI-IAD—immune checkpoint inhibitor-isolated adrenocorticotropic hormone deficiency, CTLA4—cytotoxic T lymphocyte antigen 4, PD-1—programmed cell death 1, PD-L1—programmed cell death ligand 1.

**Table 2:** *Framework characteristics*.

Citation	Age/Race/Gender/PMH-DM Host Characteristics	Underlying Malignancy Host Characteristics	Agent Characteristics	HLA Expression and T-Cell Upregulation Microenvironment	Pancreatic Auto antibodies Microenvironment	C-Peptide Microenvironment	**Corresponding** **Reference**
Stamatouli et al., 2018	◦Age: 66 years ◦Race: 24 Whites, 1 Asian, 1 Hispanic, 1 Non-Hispanic ◦Gender: 17 males, 10 females PMH-DM: 4 with pre-DM and 2 with T2DM	◦14 Melanoma ◦3 RCC ◦4 NSCLC ◦1 SCLC ◦1 CholangioCA ◦1 Neuroendocrine tumor ◦1 Pancreatic CA ◦1 GI CA ◦1 SCC	PD-1 ◦14 PD-L1 ◦1 CTLA-4 ◦None Combo ICI and Medication ◦12 Combo ICI and Chemo ◦None	HLA: None of the subjects expressed the T1DM protective allele HLA-DR2. There was a predominance of HLA-DR4 (16/21, 76%) T-Cell Upregulation --	GAD+ (9 pts), Anti IA2 (5 pts), Anti ZnT8 (2 pts), Islet cell antibodies (2 pts)	23 low or undetectable C-peptide	[28]
Kotwal et al., 2019	◦Age: 61.3 years ◦Race: 20 Whites, 1 Black ◦Gender: 12 males, 9 females PMH-DM: 11 (pre-DM or prior T2DM)	◦9 Melanoma ◦5 Lung CA ◦2 Breast CA ◦1 RCC ◦1 MM ◦1 Lymphoma ◦1 Merkel Cell CA ◦1 Esophageal CA ◦1 Pancreatic CA	PD-1 ◦21 (17 with Pembro and 4 with Nivo) PD-L1 ◦None CTLA-4 ◦None Combo ICI and Medication ◦None Combo ICI and Chemo ◦None	--	GAD+ (10/14 pts), IA-2+ (2/12 pts), ZnT8 (0/8),	Low in 10/14 pts	[41]
Marchand et al., 2019	◦Age: 67 years ◦Race: 100% White ◦Gender: 5 males, 1 female PMH-DM: None	◦(3) Melanoma ◦(1) Lung CA ◦(1) Pleiomorphic pulmonary CA ◦(1) Cutaneous T-cell lymphoma	PD-1 ◦4 PD-L1 ◦1 CTLA-4 ◦None Combo ICI and Medication ◦1 Combo ICI and Chemo ◦None	HLA: None with HLA Class II profiles (high-risk T1DM) (2) with HLA Class II haplotypes that confer protection against T1DM. T-Cell Upregulation --	GAD neg, IA-2A+ (1 pt), ZnT8 neg,	4 with undetectable C-peptide and 2 with detectable levels	[30]
TsangVHM et al., 2019	◦Age: 62 years ◦Race: NR ◦Gender: 9 males, 1 female PMH-DM: 1	Melanoma	PD-1 ◦6 PD-L1 ◦None CTLA-4 ◦None Combo ICI and Medication ◦4 Combo ICI and Chemo ◦None	HLA: 3 patients with risk haplotype for T1DM and 3 with HLA haplotype associated with protection against T1DM. T-Cell Upregulation --	Negative for islet antigen, insulin antibody, zinc transporter.	All with low C-peptide	[42]
Byun et al., 2020	◦Age: 63.5 years ◦Race: NR ◦Gender: 10 males, 8 females PMH-DM: None	The most common primary cancer was melanoma	PD-1 ◦12 PD-L1 ◦5 CTLA-4 ◦None Combo ICI-Medication ◦Nine patients received CTLA-4 and PD-1/PD-L1 blockade and one who received PD-1 and PD-L1 at different time points. Combo ICI and Chemo ◦None	HLA: HLA class I T-Cell Upregulation --	Five of the 12 patients were positive for GAD65 autoantibodies.	Of the 18 patients, 11 trended toward or had undetectable C-peptide levels.	[35]
Knight et al., 2021	◦ Age: 72 years ◦ Race: NR ◦Gender: 100% males PMH-DM: NR	◦2 with melanoma ◦1 RCC ◦1 Prostate CA	PD-1 ◦4 PD-L1 ◦None CTLA-4 ◦None Combo ICI and Medication ◦None Combo ICI and Chemo ◦None	--	--	--	[29]
Leiter 2021et al.,	◦Age: 63.9 years ◦Race: 16 Whites, 4 Blacks, 9 Hispanics, 8 Asians, 11 Unknown ◦Gender: 33 males PMH-DM: 19	◦(12) NSCLC ◦(13) HCC ◦(12) RCC ◦(17) SCC of HandN ◦(6) Melanoma ◦(4) Urothelial Cell CA ◦(4) MM ◦(3) Other	PD-1/PD-L1 ◦34 (study grouped this category with PD-1 and PD-L1) CTLA-4 ◦6 Combo ICI and Medication ◦None Combo ICI and Chemo ◦None	--	GAD+ in one patient	C-peptide low in one patient	[37]
Basak et al., 2022	◦Age: 69.5 years ◦Race: White 100% ◦Gender: 62.5% males, 37.5% females PMH DM: None	◦Melanoma and NSCLC (37.5% each). ◦RCC 12.5% ◦Urothelial Cell CA 12.5%	PD-1 ◦Nivolumab monotherapy (62.5%) ◦Pembrolizumab monotherapy (25%) PD-L1 ◦None CTLA-4 ◦None Combo ICI and Medication ◦12.5% Combo ICI and Chemo ◦None	--	Two patients (25%) in the ICI-related diabetes group had positive islet autoantibodies before the onset of ICI-DM, one at baseline and one in the last sample before the onset of ICI-related diabetes, whereas one patient (6%) in the control group had positive islet autoantibodies at baseline. After the onset of ICI-DM, four patients (50%) (of whom two were already seropositive before diabetes onset) had positive islet autoantibodies.	At baseline, the median C-peptide concentration in cases was 1.73. Two out of five patients in whom C-peptide was measured during routine clinical care had C-peptide concentrations below the reference range at diagnosis of diabetes, of whom both had diabetic ketoacidosis. The only other patient with diabetic ketoacidosis had a C-peptide level in the reference range at diagnosis.	[22]
Inaba et al., 2022	◦Age: 72.2 years ◦Race: 100% Asian ◦Gender: 6 males, 1 female PMH-DM: 1	◦4 NSCLC ◦1 metastatic melanoma in	PD-1 ◦6 PD-L1 ◦1 CTLA-4 ◦None Combo ICI and Medication ◦ 1 Combo ICI and Chemo ◦None	HLA: HLA-DRB1*04:05 allele frequencies were significantly higher in ICI-T1DM patients than in controls. Notably, the frequencies of HLA-DPA1*02:02 and its associated allele, DPB1*05:01, were significantly associated with an increased risk of ICI-T1DM compared to the controls. T-Cell Upregulation --	Pancreatic beta-cell autoantibodies were all negative except for 1 patient.	Undetectable in all patients 1 month after diagnosis	[26]
Muniz et al., 2022	◦Age: 60.5 years ◦Race: NR ◦Gender: 25 males, 9 females PMH-DM: 7 with either pre-DM or NIDDM	◦19 Melanoma ◦4 with RCC ◦4 with NSCLC ◦7 other	PD-1/PD-L1 ◦20 but combined with PD-1 and PD-L1 CTLA-4 ◦None Combo ICI and Medication ◦None Combo ICI and Chemo ◦5	--	GAD (measured in 11 pts; + in 5 pts), insulin antibodies (measured in 1 patient; neg); anti-islet cell, negative; zinc transporter, negative	Measured in 17 pts (7 with undetectable values and the another 6 although detectable was low, and 4 with normal levels.	[32]
Chan et al., 2023	◦Age: 62.2 years ◦Race: NR ◦Gender: 3375 Total, 65.2% males 326 males developed ICI-DM and 131 females developed ICI-DM PMH-DM: None	Almost half of the patients had lung CA	PD-1 ◦2749 PD-L1 ◦691 CTLA-4 ◦369 Combo ICI and Medication ◦NR Combo ICI and Chemo ◦2038	--	--	--	[27]
Inaba et al., 2023	◦Age: 75 years ◦Race: Asian 100% ◦Gender: 9 males, 3 females PMH-DM: None	◦7 NSCLC ◦3 MM ◦1 RCC ◦1 SCLC	PD-1 ◦10 PD-L1 ◦1 CTLA-4 ◦None Combo ICI and Medication ◦1 Combo ICI and Chemo ◦None	HLA: HLA-DPB1*05:01 allele frequency was more significantly associated with an increased risk of ICI-T1DM compared with general controls. T-Cell Upregulation --	--	--	[25]
Iwamoto et al., 2023	◦Age: 69 years ◦Race: NR ◦Gender: 68% males, 32% females PMH-DM: 3	Solid tumors were mentioned, but the ones associated with the development of ICI-DM were not clearly identified.	PD-1 ◦351 (1.4% with ICI-DM) PD-L1 90 (none developed ICI-DM) CTLA-4 ◦None Combo ICI and Medication ◦25 Combo ICI and Chemo ◦None	--	GAD+ in 1 patient	--	[33]
Jeun et al., 2023	◦Age: 60 years ◦Race: 63 Whites, 5 Hispanics, 4 Asians, 4 Blacks ◦Gender: 43 males PMH-DM: 12	◦23 MM ◦12 lung CA ◦11 with RCC; Others with GBM, prostate, angiosarcoma, cancer of unknown primary, HCC, parotid CA, ovarian CA, ATC, esophageal CA, and serous adeno CA	PD-1 ◦54 PD-L1 ◦11 CTLA-4 ◦1 Combo ICI and Medication ◦10 Combo ICI and Chemo ◦37% tyrosine kinase or VEGF inhibitors, or other agents intended to enhance the antitumor immune response by targeting CD137, CXCR4, IDO1, CSF1R, STAT3, or CCR4	--	GAD 65+ (40/69 pts) IA2+ (7/58 pts) Anti Insulin+ (11/58 pts) ZnT8+ (2/20 pts)	Median C-peptide at 4 weeks 0.2 ng/mL f/b 0 ng.ml	[36]
Kawata et al., 2023	◦Age: 64 years ◦Race: NR ◦Gender: 100% males PMH-DM: NR	Urothelial CA Hodgkin lymphoma RCC	PD-1 ◦2 PD-L1 ◦None CTLA-4 ◦None Combo ICI and Medication ◦1 Combo ICI and Chemo ◦None	HLA: DRAB1-DQB1+ (2 pts) T-Cell Upregulation --	GAD+ (2 pts) IA2+ (1 pt)	<0.02–0.6	[24]
Lee et al., 2023	◦Age: 60.4 years ◦Race: 100% Asian ◦Gender: 136 males PMH-DM: None	Lung, liver, breast	PD-1 ◦154 PD-L1 ◦67 CTLA-4 ◦None Combo ICI and Medication ◦None Combo ICI and Chemo ◦None	--	--	--	[23]
Liu et al., 2023	◦Age: 57.2 years ◦Race: Asian 100% ◦Gender: 17 males, 5 females PMH-DM: None	◦43.5% lung cancer ◦8.7% tongue carcinoma ◦8.7% had gastric carcinoma; the remaining were affected by different types of carcinomas	PD-1 ◦18 PD-L1 ◦4 CTLA-4 ◦None Combo ICI and Medication ◦1 Combo ICI and Chemo ◦None	HLA: 13 with HLA haplotype, 22.7% had both susceptible and protective haplotypes, and 31.8% carried only protective HLA haplotypes T-Cell Upregulation --	IAA Negative, IA-2 Negative, ZnT8 Negative, GAD+ (2 pts)	23 patients with low C-peptide	[39]
Zhang et al., 2023	◦Age: 65 years ◦Race: 22 Whites, 3 Blacks ◦Gender: 14 males, 11 females PMH-DM: 11	◦8 Melanoma ◦7 Lung CA ◦5 RCC ◦2 Bladder CA ◦1 Anal CA ◦1 Head and Neck CA ◦1 Prostate CA	PD-1 ◦16 PD-L1 ◦2 CTLA-4 ◦None Combo ICI and Medication ◦3 and an additional 4 with sequential ICI therapy Combo ICI and Chemo ◦ None	--	GAD+ in 2/11, ZnT8 (0/11), IAA (1/6)	5/11 with low C-peptide	[38]
Akturk et al., 2024	◦Age: 53.2 years ◦Race: NR ◦Gender: 62 males, 27 females PMH-DM: None	Advanced melanoma (stage III unresectable and stage IV)	PD-1 ◦Did not clearly quantify, but mentioned that 50% developed ICI-DM after anti-PD-1 PD-L1 ◦None CTLA-4 ◦None Combo ICI and Medication ◦Did not clearly quantify, but mentioned 50% developed ICI-DM after combo PD-1/PD-L1 + CTLA4 Combo ICI and Chemo ◦None	HLA: (70%) ICI-T1D patients had HLA-DR4 T-Cell Upregulation --	1 (14%) GAD+ pre-treatment and 4 GAD + post treatment	3 with C-peptide levels very low or absent	[34]
Elshafie et al., 2024	◦Age: 56 years ◦Race: NR ◦Gender: 59 males, 80 females PMH-DM: None	◦33 Breast ◦31 Lung ◦17 gastric	PD-1 ◦120 PD-L1 ◦19 CTLA-4 ◦Not reported Combo ICI and Medication ◦NR Combo ICI and Chemo ◦NR	--	1 patient GAD positive 3 months after diagnosis.	1 patient with low C-peptide 3 months after diagnosis	[40]
Ono et al., 2024	◦Age: 64.7 years ◦Race: 100% Asian ◦Gender: 21 males, 12 females PMH-DM: NR	◦10 NSCLC ◦4 RCC ◦2 Melanoma ◦2 Ureteral ◦2 Breast ◦1 Pancreatic ◦1 Oropharyngeal ◦1 Colon ◦1 Malignant Pleural Mesothelioma	PD-1 ◦22 PD-L1 ◦5 CTLA-4 ◦None Combo ICI and Medication ◦6 Combo ICI and Chemo ◦None	HLA: DRB1*09:01 − DQB1*03:03 = 27.3% DRB1*13:02 − DQB1*06:04 = 13.6% DRB1*15:02 − DQB1*06:01 = 0% T-Cell Upregulation --	(9 +) with islet autoantibodies and (7) GAD+; IA-2 (2 positive), 11 tested for ZnT8 (all negative)	--	[31]
Ruiz-Esteves et al., 2024	◦Age: 64 years ◦Race: 60 Whites, 3 Asians, 1 Black ◦Gender: 32 males, 32 females PMH DM: 16	◦23 Melanoma ◦15 Thoracic CA ◦9 GU CA ◦5 Breast CA ◦3 GI, H&N, Neuro CA ◦2 Hematological CA ◦1 CA of Unknown Primary	PD-1 ◦43 PD-L1 ◦4 CTLA-4 ◦2 Combo ICI and Medication ◦15 Combo ICI and Chemo ◦None	--	1 antibody (the study did not identify which antibodies)	38 with low C-peptide	[13]

Note: Abbreviations: NR—not reported, CA—cancer, ICI—immune checkpoint inhibitor, PD-L1—programmed death-ligand 1, PD-1—programmed cell death protein 1, CTLA-4—cytotoxic T-lymphocyte associated protein 4, MM—multiple myeloma, SCLS—small cell lung cancer, GI—gastrointestinal, SCC—squamous cell carcinoma, HCC—hepatocellular carcinoma, ATC—anaplastic thyroid carcinoma, GBM—glioblastoma, H&N—head and neck, NSCLC—non-small cell lung cancer, Chemo—chemotherapy, RCC—renal cell carcinoma, IAA—insulin autoantibodies, GAD65—glutamic acid decarboxylase 65, IA-2—islet antigen-2, ZnT8—zinc transporter 8, Pre-DM—pre-diabetes mellitus, T2DM—type 2 diabetes mellitus, PMH—past medical history, DM—diabetes mellitus.

## Data Availability

The original contributions presented in this study are included in the article. Further inquiries can be directed to the corresponding author.

## Abbreviations

The following abbreviations are used in this manuscript:

NR	NR-Not Reported
CA	Cancer
ICI	Immune Checkpoint Inhibitor
PD-L1	Programmed Death-Ligand 1
PD-1	Programmed Cell Death Protein 1
CTLA-4	Cytotoxic T-Lymphocyte-Associated Protein 4
MM	Multiple Myeloma
SCLS	Small Cell Lung Cancer
GI	Gastrointestinal
SCC	Squamous Cell Carcinoma
HCC	Hepatocellular Carcinoma
ATC	Anaplastic Thyroid Carcinoma
GBM	Glioblastoma
H&N	Head and Neck
NSCLC	Non-Small Cell Lung Cancer
Chemo	Chemotherapy
RCC	Renal Cell Carcinoma
IAA	Insulin Autoantibodies
GAD65	Glutamic Acid Decarboxylase 65
IA-2	Islet Antigen-2
ZnT8	Zinc Transporter 8
Pre-DM	Pre-Diabetes Mellitus
T2DM	Type 2 Diabetes Mellitus
PMH	Past Medical History
DM	Diabetes Mellitus
ICI-DM	Immune-Checkpoint Inhibitor diabetes mellitus
RA	Retrospective Analysis
PA	Prospective Analysis
W	Weeks
M	Months
D	Days
C	Cycles
T1DM	Type 1 Diabetes Mellitus
NR	Not Reported
CC	Conventional Chemotherapy
PTS	Patients
irAE	Immune-Mediated Adverse Effects
HLA	Human Leukocyte Antigen
ICI-IAD-	Immune Checkpoint Inhibitor Isolated Adrenocorticotropic Hormone Deficiency
CINAHL	Cumulative Index to Nursing and Allied Health Literature
MESH	Medical Subject Headings
